# Imidacloprid Movement into Fungal Conidia Is Lethal to Mycophagous Beetles

**DOI:** 10.3390/insects11080496

**Published:** 2020-08-03

**Authors:** Robin A. Choudhury, Andrew M. Sutherland, Matt J. Hengel, Michael P. Parrella, W. Douglas Gubler

**Affiliations:** 1 School of Earth, Environmental, and Marine Sciences, University of Texas Rio Grande Valley, Edinburg, TX 78539, USA; robin.choudhury@utrgv.edu; 2 University of California Cooperative Extension, Alameda County, Hayward, CA 94544, USA; 3 Department of Environmental Toxicology, University of California, Davis, Davis, CA 95616, USA; mjhengel@ucdavis.edu; 4 Department of Entomology, Plant Pathology and Nematology, University of Idaho, Moscow, ID 83844, USA; mpp@uidaho.edu; 5 Department of Plant Pathology, University of California, Davis, Davis, CA 95616, USA; wdgubler@ucdavis.edu

**Keywords:** *Psyllobora vigintimaculata*, *Podosphaera xanthii*, powdery mildew, imidacloprid, neonicotinoid, tri-trophic interaction, non-target effect

## Abstract

**Simple Summary:**

Some insects are beneficial to plants because they eat pest insects and disease-causing fungi; integrating the use of these insects into pest management can help to reduce the need for costly pesticide applications. Twenty-spotted ladybeetles eat plant pathogenic fungi, which helps to reduce disease severity for many economically important crops. In this study, we applied a systemic insecticide to the roots of pumpkin plants and monitored to see if it would be detectable in the spores of a plant pathogenic fungus and whether the insecticide-tainted fungal spores would hurt the ladybeetle larvae. We were able to chemically detect the systemic insecticide in the fungal spores up to 21 days after the plants had been treated with the fungus. We found that the ladybeetles raised on infected plants that had been treated with the systemic insecticide died more rapidly that ladybeetles that had been raised on uninfected or untreated plants. This study is the first to show that systemic insecticides can move from the roots of a plant, into a plant pathogenic fungus, and then have negative effects on a fungus-eating insect. It suggests that growers and land managers need to carefully consider the unintended consequences of insecticide applications.

**Abstract:**

Applications of systemic pesticides can have unexpected direct and indirect effects on nontarget organisms, producing ecosystem-level impacts. We investigated whether a systemic insecticide (imidacloprid) could be absorbed by a plant pathogenic fungus infecting treated plants and whether the absorbed levels were high enough to have detrimental effects on the survival of a mycophagous beetle. Beetle larvae fed on these fungi were used to assess the survival effects of powdery mildew and imidacloprid in a factorial design. Fungal conidia were collected from treated and untreated plants and were tested for the presence and concentration of imidacloprid. The survival of beetles fed powdery mildew from imidacloprid-treated leaves was significantly lower than that of the beetles from all other treatments. Imidacloprid accumulated in fungal conidia and hyphae was detected at levels considered lethal to other insects, including coccinellid beetles. Water-soluble systemic insecticides may disrupt mycophagous insects as well as other nontarget organisms, with significant implications for biodiversity and ecosystem function.

## 1. Introduction

Neonicotinoid insecticides, which disrupt insect nicotinic acetylcholine receptors, are commonly employed worldwide within pest management programs [1]. Imidacloprid, the first commercially successful neonicotinoid, is labeled for use against many phytophagous pests in agricultural and urban landscapes, such as piercing/sucking insects, bark burrowers, and chewing beetle larvae, but its activity is evident on a wide range of arthropods [2]. Direct toxicity has been shown not only to affect common target pests such as aphids and whiteflies [3], but also to negatively impact beneficial insects such as coccinellid beetles [4,5], hymenopteran parasitoids [6], and predatory mites [7]. A growing body of literature exists documenting the direct and indirect effects of imidacloprid and other neonicotinoids against pollinator insects [8,9]. Guttation fluids from treated plants have also been identified as routes to exposure for nontarget arthropods [10]. Indirect toxicity to beneficial insects may also readily occur. It has been shown that coccinellid predators may ingest lethal doses of imidacloprid through the consumption of sessile homopteran prey that have ingested this systemic material through phytophagy [11]. Even the consumption of honeydew from homopterans feeding on treated plants can negatively impact the fecundity and survival of beneficial insects [12]. Imidacloprid and its plant metabolites move readily within plants, within the environment, and through various organisms at different trophic levels, imparting toxicity to susceptible insects [1].

Plant pathogenic fungi that utilize plant water and nutrients, such as powdery mildews (Erysiphales), may act as reservoirs for systemic insecticides, and therefore may be toxic to susceptible arthropods if consumed. The cosmopolitan coccinellid tribe Halyziini is known to obligately consume Erysiphales conidia and hyphae, providing ecosystem services in natural and agricultural settings throughout the world [13]. Using this model system, and based on previous observations, we sought to determine whether imidacloprid can move into fungal hyphae and whether this movement would negatively affect the survival beetles that fed on the contaminated hyphae.

## 2. Materials and Methods

Adults of the halyziine coccinellid beetle *Psyllobora vigintimaculata* (Coleoptera: Coccinellidae: Halyziini) were collected from a vineyard in Fresno, CA, in Fall 2011. The beetles were reared over three generations (approximately 90 days) in a growth chamber (PGR-15, Conviron Ltd., Winnipeg, Canada) that was kept at 25 °C and 50% relative humidity under fluorescent lights with a 14 h photoperiod on pumpkin plants (*Cucurbita pepo* cv. Sorcerer) infected with the cucurbit powdery mildew pathogen, *Podosphaera xanthii* (syn. *P. fusca*). To encourage a uniformly aged population for our bioassay, we introduced approximately 400 beetles of mixed sex into a separate growth chamber containing pumpkin plants infected ten days prior. After four days of egg deposition, the adults were removed and the eggs left to hatch. Concurrently, the pumpkin seeds were planted into five 6 inch pots in trays and flood irrigated with either an imidacloprid solution (Admire 2F, 21.4% imidacloprid, label rate for greenhouse vegetables: 1.4 fluid oz / 21 gallons water, EPA Registration# 264-758) or deionized water in a greenhouse. After ten days, one tray each of the imidacloprid-treated and untreated plants was removed from the greenhouse, inoculated with *P. xanthii* (by gently transferring conidia from the infected leaves of other plants), and placed into a growth chamber (25 °C, 50% RH). The other two trays were kept uninoculated in the greenhouse (20 ± 10 °C, 50% ± 20% RH).

Fifteen days later, the leaves from all four seedling groups were removed and cut petioles inserted into 2 mL glass vials with deionized water, sealed with parafilm. These vials were then placed into inverted transparent plastic quart (~950 mL) containers and sealed. One beetle larva (first or second instar, due to the 4 d egg-deposition range) was randomly selected from the egg deposition chamber and introduced to each container. The containers with uninfected leaves (without powdery mildew) were supplemented with one 15 mm leaf disk cut from untreated infected plants, to provide a food source for *P. vigintimaculata* larvae, known as obligate mycophages [14]. The excised leaves and vials were changed every four days to prevent wilting; supplemental infected leaf disks were changed every three days. In this way, the treatments included: (1) untreated and uninfected with infected leaf disc supplement: IM−/PM−, (2) untreated and infected with powdery mildew: IM−/PM+, (3) imidacloprid-treated and uninfected, with infected leaf disc supplement: IM+/PM−, and (4) imidacloprid-treated and infected with powdery mildew: IM+/PM+. Each treatment was represented by 15 replicate containers, for a total of 60 bioassay arenas (see Figure S1). Containers were maintained at ambient room temperature (22 °C) and a 12 h photoperiod. The mortality, moribundity, and developmental stage were assessed for the beetle larvae daily until the emergence of adults from pupae. Moribund larvae that were immobile for two or more days were judged dead and removed from the assay. Observations continued until all the larvae were dead or had successfully pupated. Conidia from both imidacloprid treated and untreated leaves were vacuum aspirated into microcentrifuge tubes and tested for imidacloprid concentration using liquid chromatography and tandem mass spectrometry (see Supplemental Materials for the specific methods used).

Survival analyses were conducted in R v. 3.5.2 using the ‘survival’ package [15]. To analyze the main effects of imidacloprid and powdery mildew treatments on survival, in addition to the interaction effects, we generated both a full regression model and performed a pairwise log rank test using Bonferroni-adjusted *p*-values [16].

## 3. Results

**Figure 1:**
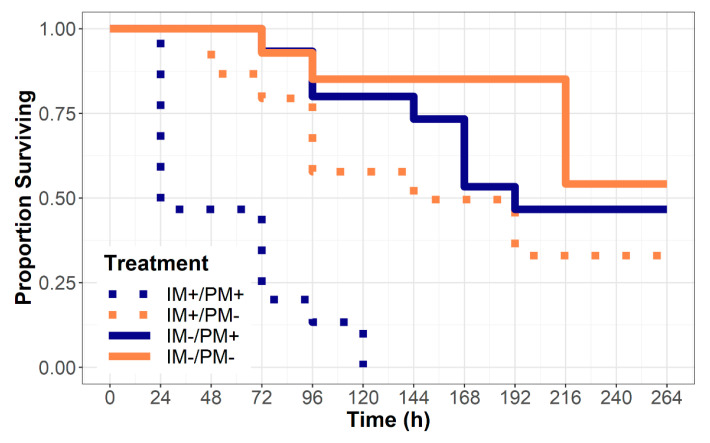
Survival of the obligately mycophagous beetle larvae, *P. vigintimaculata,* after being confined to leaves from plants either treated or untreated with imidacloprid (IM+/IM−) and either infected or uninfected by powdery mildew fungus *Podosphaera xanthii* (PM+/PM−). The larvae confined to uninfected leaves (PM−) were regularly provided sustenance by way of supplemental leaf discs from infected and untreated plants.

The qualitative examination of the survival curves suggests that the treatment groups began to diverge by ~24 h post exposure (Figure 1), and that 100% of the beetles raised on powdery mildew from imidacloprid-treated leaves died before the end the study. Survival regression, including imidacloprid treatment, powdery mildew treatment, and the interaction between the two as explanatory factors, revealed that the effects of imidacloprid treatment was substantially higher (hazard ratio (HR) = 3.66, 95% confidence interval (CI): 1.86–7.18) than the effect of powdery mildew food availability (HR = 2.18, 95% CI: 1.13–4.19). We also found a strong interaction between the imidacloprid treatment and the presence of powdery mildew (HR = 4.99, 95% CI: 1.23–20.21).

**Figure 2:**
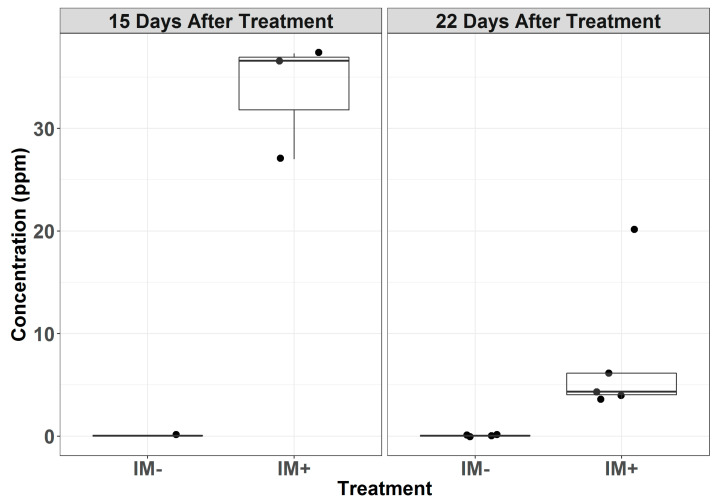
Concentration (ppm) of imidacloprid in powdery mildew conidia collected from treated (IM+) and untreated (IM−) plants 15 and 22 days after treatment. Points represent the individual samples, and the overlaying boxplots represent the quantiles.

The chemical analysis detected increased levels of imidacloprid in the conidial samples taken from the leaves excised from plants treated with imidacloprid (Tables S1–S3). Powdery mildew conidia collected from the leaves treated 15-days prior had mean imidacloprid concentrations of 33.6 µg/g (*n* = 3), whereas the conidia from untreated leaves exhibited concentrations below the detectable level (0.05 µg/g) (*n* = 1) (Figure 2). The conidia collected from the leaves 22 days after the imidacloprid treatment exhibited a mean concentration of 7.65 µg/g (*n* = 5), while the untreated leaves continued to have concentrations below the detectable level (*n* = 4).

## 4. Discussion

This study represents the first record of the tri-trophic movement of imidacloprid from plant to fungus to insect, ultimately creating the significant indirect mortality of a nontarget organism and potentially disrupting a pathway for the biological control of an important group of pathogens. We observed that all the beetle larvae fed on powdery mildew grown on imidacloprid-treated plants perished within 120 h. In contrast, the larvae confined to imidacloprid-treated leaf arenas but regularly provided with leaf discs from untreated infected plants exhibited no significant differences in survival as compared to those fed on powdery mildew grown on untreated plants. These observations suggest that the insecticide did not impact the survival of larvae through volatilization from treated leaves nor through incidental phytophagy. Instead, mortality was strictly associated with the consumption of fungal structures growing from treated plant tissue. This conclusion is supported by previous observations that soil-applied imidacloprid is readily translocated into various tissues of *C. pepo* plants [17] and that the lethal effects of imidacloprid consumption on three different coccinellid species have been observed at 6.03 µg/g [18]. In general, imidacloprid is considered very toxic to coccinellid larvae, with lethal residues at concentrations as low as 2.6 µg/g [19] and lethal contact doses as low as 1.7 µg/g [20]. The levels of imidacloprid detected in the fungal conidia in our study exceeded all of these limits, even several weeks after the initial application of the insecticide material.

Mycophagous beetles may play an important role in management and detection of powdery mildew diseases globally, and the reductions in their populations might exacerbate powdery mildew outbreaks in susceptible crops. Sutherland and Parrella [21] found that a single larvae of *P. vigintimaculata* removed an average of 6.3 cm^2^ of leaf area affected by powdery mildew during its development from egg to adult. Furthermore, Peduto et al. [22] found that the incidence of *P. vigintimaculata* adults was positively correlated with the incidence of early season grapevine powdery mildew disease, suggesting a possible use of *P. vigintimaculata* as a bioindicator for disease early in the season when disease control was most critical. The use of mycophagous beetles in greenhouse settings to directly consume powdery mildew or as early indicators of disease may help the direct management of diseases in such controlled settings [23]. Mycophagous beetles from the Halyziini are observed throughout the world in association with powdery mildew infections [13], suggesting that the potential for the indirect mortality of mycophagous beetles after exposure to systemic insecticides is widespread. Beetles from the tribe Halyziini consume powdery mildew on multiple host crops [13]; thus, the application of imidacloprid on one crop may negatively impact powdery mildew disease control across many different crops in a mixed agricultural landscape. Mitigating such effects requires the coordination and cooperation across multiple stakeholders.

The ecosystem-level impacts of direct and indirect exposure to lethal doses of systemic insecticides is still being explored. Several studies have explored the tri-trophic movement of systemic insecticides and the subsequent disruption of natural predators and parasitoids [4,6,7,10,11]. While some studies demonstrated the lethal impacts of the direct applications of pesticides to mycophagous beetles [4,24], our study showed that indirect exposure to imidacloprid through the fungal food source can also rapidly lead to death in mycophagous beetles. Systemic insecticides like imidacloprid also result in reductions to fecundity and alterations of behavior in beneficial insects [6,25], impacting the sustainability and persistence of threatened populations. Widespread measures of reductions in insect diversity and abundance have recently been attributed to intensive agricultural activities and associated pesticide inputs [26]; it is possible that the trophic movement of water-soluble toxins, as observed in our study, play a part in this global problem.

## 5. Conclusions

Systemic insecticides play a critical role in disrupting integrated pest management, through both direct and indirect effects on beneficial insects. In this study, we showed that imidacloprid could move systemically through a plant into the hyphae and conidia of a plant pathogenic fungus, and the transported chemical can directly affect the survival of mycophagous beetles feeding on that fungus. This disruption of the survival of mycophagous beetles can affect both the use of these beetles to reduce the total amount of fungal conidia produced on affected plants, as well as potentially impact the use of these beetles as bioindicators of disease presence. Imidacloprid persisted in fungal conidia for several weeks after application and may present a persistent threat to other beneficial insects that are opportunistically mycophagous. The effects of systemic insecticides on fecundity and behavior may also impact the success of these insects in controlling plant disease epidemic outbreaks. Future work to explore the direct and indirect effects of systemic insecticides at the landscape level will help to clarify how these tools impact integrated pest management practices and the populations of beneficial insects.

## Supplementary Materials

The following are available online at https://www.mdpi.com/2075-4450/11/8/496/s1, Table S1: Compound specific information for the chromatography and mass spectrometry conditions. Table S2: Average imidacloprid recoveries from powdery mildew hyphae. Table S3: Non-parametric comparisons for each treatment pair using the Wilcoxon method. Asterisks represent the significance at the Bonferroni adjusted *p*-value of 0.0083. Figure S1: Experimental setup of a portion of the imidacloprid-*Psyllobora* bioassay, including detached cucurbit leaves inside of the sealed polyethylene containers and *Psyllobora* beetles on the surface of the leaves.

## References

[1] Bonmatin J.-M., Giorio C., Girolami V., Goulson D., Kreutzweiser D.P., Krupke C., Liess M., Long E., Marzaro M., Mitchell E.A.D. (2015). Environmental fate and exposure; neonicotinoids and fipronil. Environ. Sci. Pollut. Res..

[2] Pisa L.W., Amaral-Rogers V., Belzunces L.P., Bonmatin J.M., Downs C.A., Goulson D., Kreutzweiser D.P., Krupke C., Liess M., McField M. (2015). Effects of neonicotinoids and fipronil on non-target invertebrates. Environ. Sci. Pollut. Res..

[3] Qu Y., Xiao D., Li J., Chen Z., Biondi A., Desneux N., Gao X., Song D. (2015). Sublethal and hormesis effects of imidacloprid on the soybean aphid *Aphis glycines*. Ecotoxicology.

[4] Lee Y.S., Jang M.J., Lee H.A., Lee J.H. (2017). Toxicity of pesticides to mycophagous ladybird, *Illeis koebelei* Timberlake (Coleoptera: Coccinellidae: Halyziini). Korean J. Pestic. Sci..

[5] Skouras P.J., Brokaki M., Stathas G.J., Demopoulos V., Louloudakis G., Margaritopoulos J.T. (2019). Lethal and sub-lethal effects of imidacloprid on the aphidophagous coccinellid hippodamia variegata. Chemosphere.

[6] Tappert L., Pokorny T., Hofferberth J., Ruther J. (2017). Sublethal doses of imidacloprid disrupt sexual communication and host finding in a parasitoid wasp. Sci. Rep..

[7] Put K., Bollens T., Wäckers F., Pekas A. (2016). Non-target effects of commonly used plant protection products in roses on the predatory mite *Euseius gallicus* Kreiter & Tixier (Acari: Phytoseidae). Pest Manag. Sci..

[8] Bonmatin J., Moineau I., Charvet R., Fleche C., Colin M., Bengsch E. (2003). A LC/APCI-MS/MS method for analysis of imidacloprid in soils, in plants, and in pollens. Anal. Chem..

[9] Goulson D. (2013). An overview of the environmental risks posed by neonicotinoid insecticides. J. Appl. Ecol..

[10] Hoffmann E.J., Castle S.J. (2012). Imidacloprid in melon guttation fluid: A potential mode of exposure for pest and beneficial organisms. J. Econ. Entomol..

[11] Grafton-Cardwell E.E., Gu P. (2003). Conserving vedalia beetle, *Rodolia cardinalis* (Mulsant) (Coleoptera: Coccinellidae), in citrus: A continuing challenge as new insecticides gain registration. J. Econ. Entomol..

[12] Calvo-Agudo M., González-Cabrera J., Picó Y., Calatayud-Vernich P., Urbaneja A., Dicke M., Tena A. (2019). Neonicotinoids in excretion product of phloem-feeding insects kill beneficial insects. Proc. Natl. Acad. Sci. USA.

[13] Sutherland A.M., Parrella M.P. (2009). Mycophagy in Coccinellidae: Review and synthesis. Biol. Control.

[14] Davidson W. (1921). Observations on Psyllobora taedata LeConte, a coccinellid attacking mildews. Entomol. News.

[15] Therneau T.M. (2015). A Package for Survival Analysis in S, R Package Version 2.38. https://cran.r-project.org/web/packages/survival/.

[16] Zar J.H. (2010). Biostatistical Analysis.

[17] Stoner K.A., Eitzer B.D. (2012). Movement of soil-applied imidacloprid and thiamethoxam into nectar and pollen of squash (*Cucurbita pepo*). PLoS ONE.

[18] Krischik V., Rogers M., Gupta G., Varshney A. (2015). Soil-applied imidacloprid translocates to ornamental flowers and reduces survival of adult *Coleomegilla maculata*, *Harmonia axyridis*, and *Hippodamia convergens lady beetles*, and *larval Danaus plexippus* and *Vanessa cardui butterflies*. PLoS ONE.

[19] Mizell R.F., Sconyers M.C. (1992). Toxicity of imidacloprid to selected arthropod predators in the laboratory. Fla. Entomol..

[20] Skouras P.J., Stathas G.J., Voudouris C.C., Darras A.I., Tsitsipis J.A., Margaritopoulos J.T. (2017). Effect of synthetic insecticides on the larvae of *Coccinella*
*septempunctata* from Greek populations. Phytoparasitica.

[21] Sutherland A.M., Parrella M.P. (2006). Quantification of powdery mildew removal by the mycophagous beetle *Psyllobora vigintimaculata* (Coleoptera: Coccinellidae). IOBC WPRS Bull..

[22] Peduto F., Sutherland A., Hand E., Broome J., Parikh P., Bettiga L., Smith R., Mahaffee W., Gubler W. (2011). Comparing the efficiency of visual scouting, spore trapping systems and a bioindicator for early detection of *Erysiphe necator* in California vineyards. Phytopathology.

[23] Parrella M.P., Lewis E. (2017). Biological control in greenhouse and nursery production: Present status and future directions. Am. Entomol..

[24] Sutherland A.M., Gubler W.D., Parrella M.P. (2010). Effects of fungicides on a mycophagous coccinellid may represent integration failure in disease management. Biol. Control.

[25] Xiao D., Zhao J., Guo X., Chen H., Qu M., Zhai W., Desneux N., Biondi A., Zhang F., Wang S. (2016). Sublethal effects of imidacloprid on the predatory seven-spot ladybird beetle *Coccinella septempunctata*. Ecotoxicology.

[26] Sánchez-Bayo F., Wyckhuys K.A. (2019). Worldwide decline of the entomofauna: A review of its drivers. Biol. Conserv..

